# Cost-Effectiveness of Focal Mass Drug Administration and Mass Drug Administration with Dihydroartemisinin–Piperaquine for Malaria Prevention in Southern Province, Zambia: Results of a Community-Randomized Controlled Trial

**DOI:** 10.4269/ajtmh.19-0661

**Published:** 2020-07-02

**Authors:** Joshua O. Yukich, Callie Scott, Kafula Silumbe, Bruce A. Larson, Adam Bennett, Timothy P. Finn, Busiku Hamainza, Ruben O. Conner, Travis R. Porter, Joseph Keating, Richard W. Steketee, Thomas P. Eisele, John M. Miller

**Affiliations:** 1 Department of Tropical Medicine, Center for Applied Malaria Research and Evaluation, Tulane University School of Public Health and Tropical Medicine, New Orleans, Louisiana;; 2 PATH Malaria Control and Elimination Partnership in Africa (MACEPA), Seattle, Washington;; 3 PATH MACEPA, Lusaka, Zambia;; 4 Department of Global Health, Boston University School of Public Health, Boston, Massachusetts;; 5 Malaria Elimination Initiative, Global Health Group, University of California San Francisco, San Francisco, California;; 6 National Malaria Control Centre, Zambia Ministry of Health, Lusaka, Zambia

## Abstract

Community-wide administration of antimalarial drugs in therapeutic doses is a potential tool to prevent malaria infection and reduce the malaria parasite reservoir. To measure the effectiveness and cost of using the antimalarial drug combination dihydroartemisinin–piperaquine (DHAp) through different community-wide distribution strategies, Zambia’s National Malaria Control Centre conducted a three-armed community-randomized controlled trial. The trial arms were as follows: 1) standard of care (SoC) malaria interventions, 2) SoC plus focal mass drug administration (fMDA), and 3) SoC plus MDA. Mass drug administration consisted of offering all eligible individuals DHAP, irrespective of a rapid diagnostic test (RDT) result. Focal mass drug administration consisted of offering DHAP to all eligible individuals who resided in a household where anyone tested positive by RDT. Results indicate that the costs of fMDA and MDA per person targeted and reached are similar (US$9.01 versus US$8.49 per person, respectively, *P* = 0.87), but that MDA was superior in all cost-effectiveness measures, including cost per infection averted, cost per case averted, cost per death averted, and cost per disability-adjusted life year averted. Subsequent costing of the MDA intervention in a non-trial, operational setting yielded significantly lower costs per person reached (US$2.90). Mass drug administration with DHAp also met the WHO thresholds for “cost-effective interventions” in the Zambian setting in 90% of simulations conducted using a probabilistic sensitivity analysis based on trial costs, whereas fMDA met these criteria in approximately 50% of simulations. A sensitivity analysis using costs from operational deployment and trial effectiveness yielded improved cost-effectiveness estimates. Mass drug administration may be a cost-effective intervention in the Zambian context and can help reduce the parasite reservoir substantially. Mass drug administration was more cost-effective in relatively higher transmission settings. In all scenarios examined, the cost-effectiveness of MDA was superior to that of fMDA.

## INTRODUCTION

Malaria vector control has been a major contributor to the substantial reduction of malaria burden in Zambia specifically, and sub-Saharan Africa generally, over the past 15 years.^1–4^ Definitive evidence of progress in rolling out interventions to affected communities and reductions in the burden of malaria is now available.^2,3^ In the context of this success, there have been calls for malaria elimination as well as an increased recognition that control and elimination strategies may need to include direct attempts to reduce the size of the parasite reservoir rather than only focus on reductions in human vector contact.^5,6^ Zambia’s Ministry of Health and its National Malaria Elimination Centre (NMEC), in collaboration with multiple partners, set high targets for intervention coverage, malaria burden reduction, and elimination in the country’s National Malaria Elimination Strategic Plan 2017–2021, including achieving malaria-free areas in several parts of the country.^7^ One of these areas is Southern Province, a place of continued transmission and also a testing ground for strategies to move Zambia toward malaria elimination.^8^

In 2012–2013, Southern Province, Zambia, was the site of a large trial of mass testing and treatment for malaria in which mass population screening with rapid diagnostic tests (RDTs) was conducted followed by treatment of those testing positive for malaria infection.^8^ This trial found modest effects on malaria prevalence (∼50% odds ratio) and clinical incidence through passive case detection (17% reduction) neither of which were statistically significant, although adjusted analyses yielded statistically significant results and similar effect sizes.^8^ Despite modest success, the impact on disease transmission was not viewed to be substantial enough to transition Southern Zambia to an aggressive case investigation strategy or to achieve elimination.

For this reason, from December 2014 to February 2016, the NMEC decided to embark on a large-scale trial of two more aggressive interventions for the purpose of both reducing the malaria burden in Southern Province, Zambia and possibly interrupting transmission. The NMEC designed and conducted a trial that compared three arms: 1) the standard of care (SoC), which included high vector control coverage with long-lasting insecticide-treated nets (LLINs), indoor residual spraying (IRS) using pirimiphos-methyl, community case management for malaria to improve access to quality diagnosis and treatment, and improved surveillance; 2) the SoC plus mass drug administration (MDA) using dihydroartemisinin–piperaquine (DHAp); and 3) SoC plus focal MDA (fMDA) with DHAp.^9^ Although MDA (the administration of an antimalarial drug at therapeutic doses to an entire population) is known to at least temporarily reduce the burden of malaria in some settings, relatively few community-randomized controlled trials of this strategy had been conducted.^10,11^ This article uses data and results of the trial combined with costing of the interventions to assess the incremental cost-effectiveness of adding an MDA or fMDA strategy to SoC malaria control in Southern Zambia.

## METHODS

Reporting of the study followed the guidelines presented in the Consolidated Health Economic Evaluation Reporting Standards statement.^12^

### Trial site.

**Figure 1.**
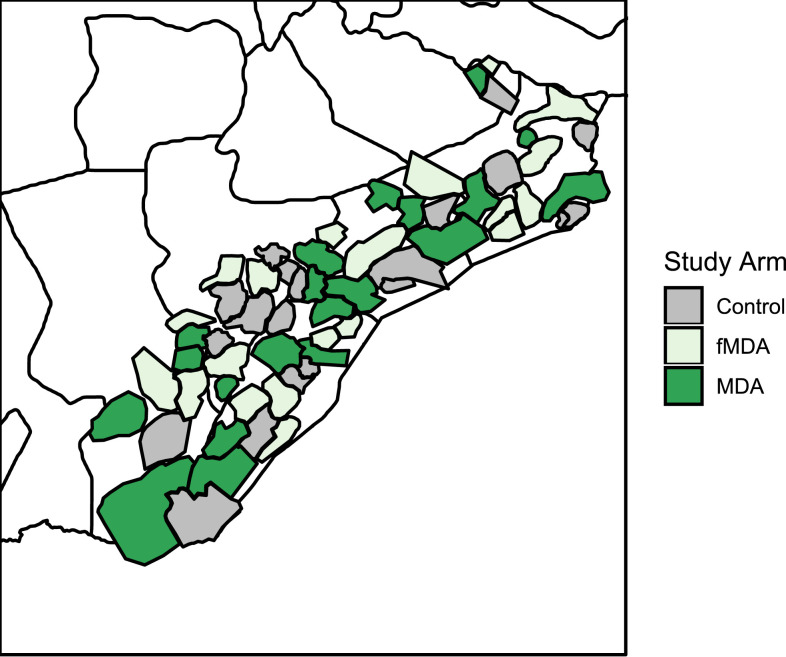
Map of Southern Province districts and health facility catchment areas included in the trial. This figure appears in color on page 8 of this issue and online at www.ajtmh.org.

Southern Province was identified in 2014 as an area where a trial of MDA/fMDA could be conducted. Parts of five districts within Southern Province were selected after having already participated in a previous mass testing and treatment trial.^8^ The districts which sit along Lake Kariba at the border of Zambia and Zimbabwe are as follows: Siavonga, Gwembe, Sinazongwe, southeastern Kalomo, and southern Choma (Figure 1). The population of the study area was estimated to be just more than 330,000 people in 2011, living in roughly 56,000 households (HH).

### Trial design.

The trial used a three-armed community-randomized controlled design. Within the five districts, 60 health facility catchment areas (HFCAs) were randomly allocated to one of three arms described previously (SoC, fMDA, and MDA) after stratification into low and high malaria transmission areas based on previous passive incidence and prevalence measurements. The details of the study design and protocol are described in the published trial protocol.^9^

### Intervention.

The control arm, SoC, consisted of high vector control coverage with LLINs and IRS, community case management, and support for facility-based diagnosis and treatment. The fMDA arm consisted of all SoC interventions plus fMDA, which in this case was conducted by population-wide screening using malaria RDTs for *Plasmodium falciparum*, followed by the treatment of all people with positive test results with a full therapeutic course of DHAp along with all members of their household, regardless of infection status. The MDA arm consisted of house-to-house visits where all consenting participants except for pregnant women and infants were given a full therapeutic course of DHAp. In both the fMDA and MDA arms, treated persons/HH were given directly observed treatment for the first DHAp dose; community health workers then returned to the household on the two subsequent days to encourage participants to fully adhere with the dosing regimen. Further details of the intervention are described in the published study protocol.^9^

### Cost collection and analysis methods.

Data on costs covered the period between 2014 and 2015, during which two rounds of MDA/fMDA were conducted during the dry season in Southern Province. An ingredient approach was used for cost estimation, where inputs were identified, valued, quantified, and classified into activity categories. Costs were classified as either capital or recurrent costs and as either traded or non-traded goods.^13,14^

Costs were initially measured in one of three currencies, Zambian kwacha, USD, or international dollars (when the source of cost info was WHO-CHOosing Interventions which are Cost-Effective (CHOICE)). Costs incurred for non-traded items procured in Zambia were first adjusted for local inflation using the Zambian Consumer Price Index reported by the Bank of Zambia, and then converted to USD using the official exchange rate.^15,16^ All other costs were first converted to USD based on official yearly average exchange rates for the period during which the costs were incurred. These costs were then adjusted for inflation to 2015 prices using the U.S. gross domestic product deflator as reported by the U.S. Bureau of Economic Analysis.^17^

Both financial and economic costs were estimated to calculate the value of donated inputs as well as the actual financial implications of the intervention. Financial costs represent purely monetary flows, whereas economic costs represent the value (or opportunity cost) of all resources necessary to implement a given intervention. However, in the case of this study, no substantial donated items were used and few capital goods were used; as such, the difference between financial and economic costs was negligible, and only economic costs are presented here. The provider perspective was used, meaning that travel or time costs to recipients of the intervention were not included, nor were other household-level costs or cost savings. Although household-level cost savings due to averted treatment could be substantial, they were beyond the scope and purpose of this evaluation and would have accrued in a greater amount to the more effective intervention arm. Household costs for the intervention were believed to be negligible, given that the intervention was provided at no charge to the household, and the drugs administered had very low risk of serious side effects that would require any medical intervention.

Because this analysis compares the two intervention arms with the SoC and each other, it focuses on incremental costs of the interventions. As a result, costs associated with existing infrastructure and other inputs that would be present without the intervention, such as the capital costs of building health facilities or training community health workers for their general roles outside of this campaign, were not included. Costs were included for inputs used at the HFCA level and the district and national levels. The capture of costs at the HFCA level used a standardized data collection form to ensure uniformity of results across all HFCAs. Tests for economies of scale were performed using linear regression. We also did not include potential cost savings to the health system because of averted treatment, given that data on these costs were not available within the scope of this study. The implications of this decision are discussed further in the discussion.

### Logistics and output data.

Data on levels of effort and outputs of the program including quantities of inputs such as DHAp treatment courses, RDT kits, vehicle days, supervision days, and other program inputs were collected directly using program records. Information on outputs including the number of persons tested and the number of persons treated was collected by direct reports from community health workers administering the intervention. Cost outcomes were examined for economies of scale by regressing these outcomes against cost using the catchment-level cost and output data, and by visually examining these relationships in scatterplots.

### Costing scenarios and sensitivity analysis.

The base-case costing scenario relied on the following set of assumptions: a discount rate of 3% was applied to capital costs; wastage of RDT kits, artemisinin combination therapy (ACT) treatment courses, and other fieldwork materials was assumed to be 10%; overhead for national and international supervision amounted to 15% of the total direct financial costs of the program. These are believed to be conservative assumptions, meaning that they would result in higher costs than that would probably occur in practice. The costs of DHAp treatment courses and RDT kits were based on the cost, insurance, and freight (c.i.f.) price of the drug or diagnostic derived from project records. Test positivity rates and population coverage estimates were assumed to be identical to those reported in the trial data set. We also conducted a sensitivity analysis by including the variability of both costs and effects as derived from the primary cost and trial effectiveness estimates in a probabilistic sensitivity analysis (PSA). In addition, a second costing of the intervention was conducted in an operational setting. The cost estimates from the operational setting were used in scenario analysis. The costing followed the principles outlined in Larson et al.^18^ Probabilistic sensitivity analysis and estimates of disability-adjusted life years (DALYs—lost years of healthy life) averted used the assumptions shown in Table 1. All parameters were assumed to be independent. Case fatality rate was assumed to be equal between infections and confirmed cases, resulting in estimates of deaths and DALYs for the case-based analysis that were smaller as they are only based on deaths and DALYs among those attending health facilities.

**Table 1:** Assumptions in probabilistic sensitivity analysis and DALY calculations

Parameter	Value
Control arm incidence of infection (high transmission)	Poisson distribution λ = 91.3 per 1,000 PY*
Control arm incidence of infection (low transmission)	Poisson distribution λ = 18.7 per 1,000 PY*
Control arm incidence of cases (high transmission)	Poisson distribution λ = 54.8 per 1,000 PY*
Control arm incidence of cases (low transmission)	Poisson distribution λ = 6.1 per 1,000 PY*
Effectiveness (relative risk) of MDA on infections (low transmission)	Lognormal distribution µ = 0.20; σ = 0.86*
Effectiveness (relative risk) of MDA on infections (high transmission)	Lognormal distribution µ = 0.41; σ = 0.45*
Effectiveness (relative risk) of fMDA on infections (low transmission)	Lognormal distribution µ = 0.63; σ = 0.73*
Effectiveness (relative risk) of fMDA on infections (high transmission (relative risk)	Lognormal distribution µ = 0.75; σ = 0.46*
Effectiveness (relative risk) of MDA on cases (low transmission)	Lognormal distribution µ = 0.50; σ = 0.18*
Effectiveness (relative risk) of MDA on cases (high transmission)	Lognormal distribution µ = 0.85; σ = 0.15*
Effectiveness (relative risk) of fMDA on cases (low transmission)	Lognormal distribution µ = 0.8; σ = 0.15*
Effectiveness (relative risk) of fMDA on cases (high transmission)	Lognormal distribution µ = 0.97; σ = 0.15*
Cost of MDA per person reached	Lognormal distribution µ = 8.49; σ = 2.48*
Cost of fMDA per person reached	Lognormal Distribution µ = 9.01; σ = 3.80*
Case fatality rate per infection or case	0.0045 ref. 32
DALY per infection or case	0.0173 ref. 33
DALY per death	33 ref. 34

DALY = disability-adjusted life year; fMDA = focal mass drug administration; MDA = mass drug administration; PY = person-years.

* Indicates that parameter values were derived from trial data.

### Outcome indicators.

Two outcome indicators were calculated: 1) cost per person reached, calculated as the total costs of the intervention divided by the total number of persons reached, and 2) cost per person treated, calculated as the total costs of the intervention divided by the number of persons treated. All outcome indicators were disaggregated to the district and HFCA levels to facilitate sub-analysis of district-level factors and HFCA-level factors associated with the cost–outcome relationship. The number of infections averted and cases averted in each arm was calculated by using the control arm incidence in each trial strata and assuming that the effect estimates (and CIs) produced in the trial for MDA and fMDA applied to the full population in each HFCA (Table 1). Infections averted were then calculated by subtracting the number of infections or cases estimated assuming MDA or fMDA was applied from the estimated number of infections or cases when no intervention was assumed.

### Impact indicators.

Two impact indicators were calculated: 1) cost per infection averted, calculated as the total costs of the intervention divided by the estimated number of infections averted based on the results of the clinical trial cohort data collection estimate for force of infection, and 2) costs per clinical case averted, calculated as the total costs of the intervention divided by the number of clinical cases averted for the entire targeted population based on the incident rate ratio estimated for exposure to the intervention in a Poisson regression model based on passive data collection in the health facilities in the trial in an earlier publication and an accompanying article.^19,20^ These indicators were also used to calculate a cost per death and DALY averted, for comparison to international thresholds and other literature, relying on simple assumptions about the case fatality rate for community-acquired infections and for cases presenting at health facilities as well as an assumption about the number of DALYs per case and per death. Uncertainty in incremental cost-effectiveness ratio (ICER) estimates were quantified using 95% confidence ellipses and through calculation of cost-effectiveness acceptability curves (Figure 4) based on the results of PSA. Assumptions used in PSA are given in Table 1.

## RESULTS

### Cost.

Although the MDA arm was slightly lower in cost per targeted person, all costs per output were statistically similar across both arms, with the exception being cost per person treated, which, because of the much more widespread treatment, was significantly lower in the MDA arm than in the fMDA arm (Table 2). Differences in cost by line item between the two interventions were almost exclusively due to the differences in the amounts of drug and or diagnostics used (i.e., more drugs in the MDA arm and more RDTs in the fMDA arm). No statistically significant differences were seen in the cost per person targeted or reached for MDA or fMDA between high and low transmission strata, but the sample size was small for each of these tests.

**Table 2:** Summary cost per output per HFCA

Output	Study arm	Mean	Interquartile Range
Total cost per HFCA	fMDA	45,638	28,708
	MDA	51,286	24,793
*P* = 0.23	All	48,462	28,413
Cost per person reached per HFCA	fMDA	8.90	5.03
	MDA	9.42	3.51
*P* = 0.55	All	9.16	4.57
Cost per household reached per HFCA	fMDA	42.52	22.82
	MDA	41.89	18.12
*P* = 0.87	All	42.20	21.96
Cost per person treated per HFCA	fMDA	85.69	39.92
	MDA	9.42	3.51
*P* < 0.0001	All	47.56	23.83

fMDA = focal mass drug administration; HFCA = health facility catchment area; MDA = mass drug administration.

Nearly 300,000 persons received the interventions during the trial at a total cost of approximately $2 million (Table 3).

**Table 3:** Total cost and population per district/arm

District	Arm	Total cost (USD)	Total population
Choma	fMDA	199,724	30,731
	MDA	188,326	25,470
Gwembe	fMDA	61,454	12,297
	MDA	181,971	20,495
Kalomo	fMDA	110,980	19,599
	MDA	61,872	11,200
Kalomo/Zimba	MDA	105,249	17,875
Mazabuka	fMDA	28,452	1,800
Mazabuka/Chikankata	fMDA	31,041	2,319
	MDA	29,859	2,351
Monze	MDA	38,974	4,077
Siavonga	fMDA	186,186	24,338
	MDA	150,757	20,907
Sinazongwe	fMDA	294,929	41,309
	MDA	268,702	34,259
Total	MDA	1,025,710	136,634
	fMDA	912,767	132,393

fMDA = focal mass drug administration; MDA = mass drug administration.

**Figure 2.**
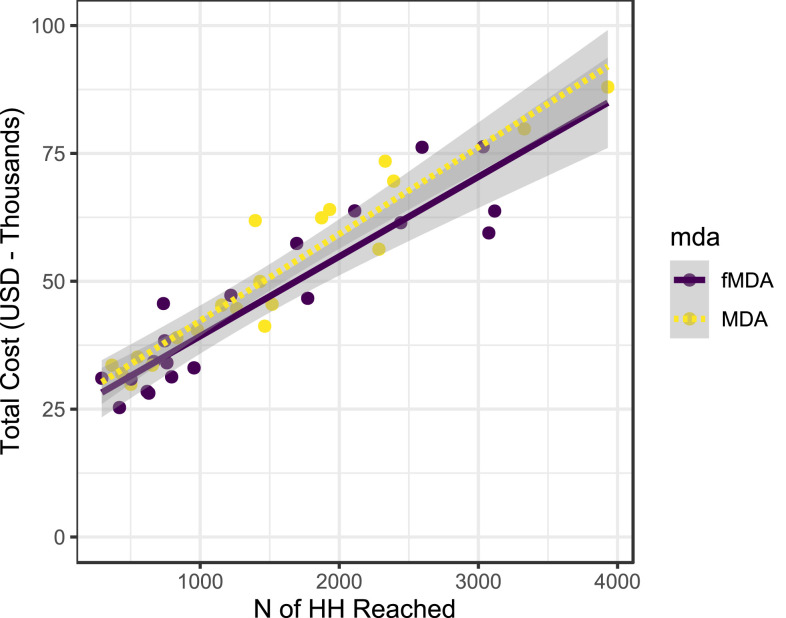
Total cost per health facility catchment area vs. households (HH) reached per health facility catchment area for focal mass drug administration (fMDA) and MDA (left) and cost vs. persons reached for fMDA and MDA (right).

To examine the data for any evidence of economies of scale, the total cost per HFCA was regressed against the total number of houses and persons reached in each trial arm. There was a direct linear relationship between these quantities and no evidence of economies of scale (Figure 2). A subsequent cost analysis during the wider scale operational implementation of MDA following the trial yielded much lower estimates of cost (US$2.90 per person reached) (K. Silumbe, personal communication).

### Cost-effectiveness.

The trial was designed to estimate the cost per infection and case averted by MDA and fMDA. Mass drug administration showed superior cost-effectiveness in terms of infections averted (Table 4) and appeared to improve when used in relatively higher infection incidence settings (as did fMDA). An analysis was also conducted based on passive case detection of symptomatic individuals at health facilities. Mass drug administration also demonstrated superior cost-effectiveness to fMDA in terms of cases averted at health facilities. Mass drug administration and fMDA were both superior in high transmission areas to their cost-effectiveness in low transmission areas in terms of cost per case averted and in terms of cost per infection averted. When comparing fMDA directly with MDA for incremental analysis, the costs per person covered were statistically indistinguishable between the two interventions; thus, the intervention that was more effective, MDA, was considered superior in all situations.

**Table 4:** Incremental cost per infection and case averted vs. standard of care

Transmission level	Study arm	Number of study clusters	Incremental cost per infection averted	Incremental cost per case averted
High	fMDA	10	429	5,951
High	MDA	10	164	1,076
Low	fMDA	10	1,119	6,755
Low	MDA	10	544	2,666
Overall	fMDA	20	810	6,353
Overall	MDA	20	354	1,872

fMDA = focal mass drug administration; MDA = mass drug administration.

### Sensitivity analysis.

**Figure 3.**
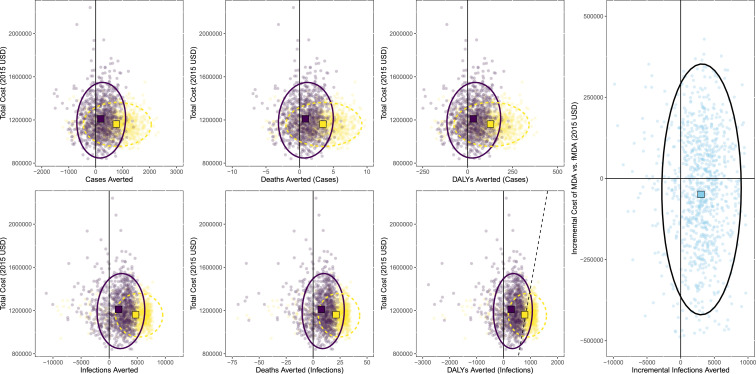
Scatterplots of cost vs. outcomes derived from probabilistic sensitivity analysis of trial results. Focal mass drug administration (fMDA) is shown in dark purple and MDA shown in light yellow; ellipses are 95% CI and are also shown in dark purple solid lines for fMDA and in light yellow dashed line for MDA (black for incremental analysis of MDA vs. fMDA). Squares represent the center of data clouds with light yellow for MDA and dark purple for fMDA. Rightmost chart is the incremental analysis of MDA compared with fMDA. Dashed black lines in disability-adjusted life year (DALY) chart represent a willingness to pay a threshold of 1,414 USD (approximately equivalent to the gross domestic product of Zambia per capita at the time of the trial). This figure appears in color at www.ajtmh.org.

**Figure 4.**
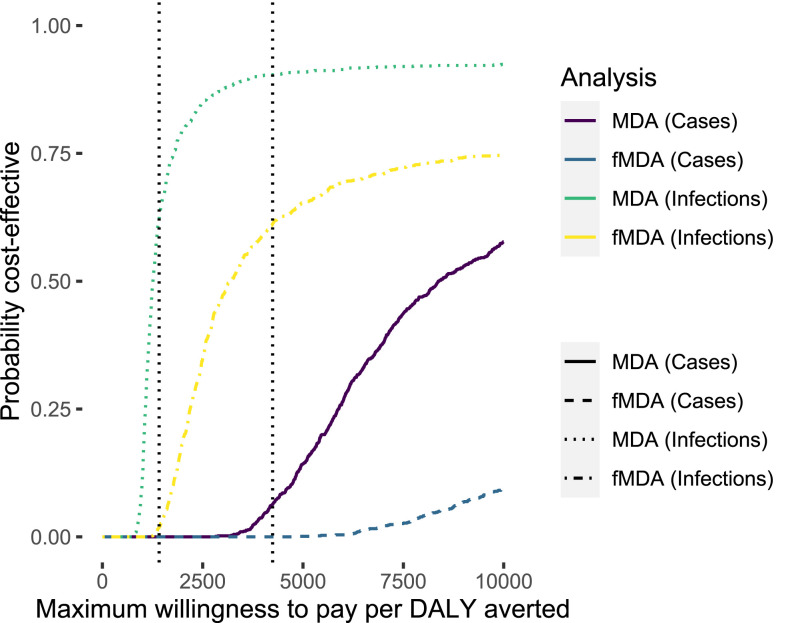
Cost-effectiveness acceptability curves for disability-adjusted life years (DALYs) averted using focal mass drug administration (fMDA) or MDA with analysis based on either infections averted—community cohort surveillance—or cases averted—passive health facility surveillance. Vertical dotted lines represent the WHO thresholds for highly cost-effective (lower willingness to pay [WTP]) and cost-effective (higher WTP) in Zambia. The probability that intervention can be considered cost-effective can be read for any WTP threshold by finding the WTP value on the *x*-axis and reading the corresponding *y*-axis value for the specific intervention and effectiveness measurement method shown in the legend. WHO thresholds are indicative for Zambia and represent 1× and 3× gross domestic product per capita in Zambia.

To understand the level of certainty surrounding these estimates, several sensitivity analyses were conducted. These included both a PSA and simple modeling of the infection and case outcomes into death and DALYs averted. There is a large amount of overlap between the estimates from the two trial arms. Similar results were seen for the cost per case averted analysis (Figure 3). These results also indicate a significant amount of overlap and show that MDA averted slightly more cases at a similar cost as fMDA. Because it is sometimes difficult to interpret data clouds when the density distributions overlap so thoroughly, cost-effectiveness acceptability curves were also constructed for these analyses (Figure 4). The results indicate that when considered in terms of outcomes based on infections averted, MDA is likely to yield a result consistent with the WHO definitions of a cost-effective intervention for Zambia. In general, MDA appears to be more likely to be a cost-effective intervention, whereas in all cases, fMDA does not appear likely to achieve WHO thresholds for being considered a “cost-effective” or “highly cost-effective” intervention.^21^ Table 5 shows the results of the sensitivity analysis in terms of cost per DALY averted (Table 5).

**Table 5:** Incremental cost per DALY averted vs. standard of care

Outcome (arm, model)	Incremental cost per DALY averted
Incremental cost per DALY (MDA, infections)	2,137
Incremental cost per DALY (fMDA, infections)	4,889
Incremental cost per DALY (MDA, cases)	11,299
Incremental cost per DALY (fMDA, cases)	38,344

DALY = disability-adjusted life year; fMDA = focal mass drug administration; MDA = mass drug administration

The cost per person reached in operational deployment was combined with the trial estimates of effectiveness as an additional scenario analysis. This reduced the cost per person treated under MDA by more than 50% and resulted in an improvement in cost-effectiveness such that MDA would be considered highly cost-effective in the Zambian context in a majority of scenarios.

## DISCUSSION

This study measured the cost-effectiveness of MDA and fMDA, compared with each other and with a SoC without any mass treatment intervention, in the context of a large community-randomized controlled trial in Southern Province, Zambia. Results indicate that MDA is superior to fMDA from a cost-effectiveness perspective. Although there was a high level of overlap in the cost-effectiveness estimates for MDA and fMDA, only MDA showed a high probability of being considered a cost-effective investment in the Zambian context.

To our knowledge, this study represents the first time that the cost-effectiveness of fMDA for malaria control and prevention has been estimated based on primary data collection. In addition, these estimates are derived from a rigorous community-randomized controlled trial, providing robust estimates of efficacy.^19,20^ As we were able to calculate costs on a per HFCA basis, we were able to study the variability not only in expected effects but also in the expected costs and incorporate these results into a PSA, strengthening our understanding of the uncertainty surrounding our estimates.

There is substantial evidence available on the cost of MDA in sub-Saharan African settings, mainly from studies of neglected tropical diseases.^22^ In malaria, there are also recent cost and effectiveness studies dedicated to a form of MDA called seasonal malaria chemoprophylaxis (SMC, formerly intermittent preventative treatment in children).^23,24^ These strategies involve targeted MDA to population subgroups, whereas our study involved distribution to all age- and gender groups, with only limited exclusions for drug safety reasons.

The costs that were shown in this trial are significantly higher than those shown in other studies of MDA in neglected tropical disease settings as well as for mass testing and treatment in the same area of Zambia.^22–25^ There are several likely explanations for this finding, including the higher cost of the drug used, the extensive transportation costs involved in the early rounds of this intervention, and the fact that the mass treatment interventions were implemented as part of a randomized controlled trial. It would be expected that cost per person targeted will fall significantly in future MDA or fMDA rounds implemented under routine program conditions. Indeed, subsequent data collection during operational implementation of MDA found costs per person that were more than 50% lower per person than those found during the trial. If such improvements in technical efficiency did not compromise the effectiveness of the intervention, the cost-effectiveness would be substantially better than what is shown in our main analysis. Cost studies of SMC have also shown significant variability in cost per course of drug administered despite finding that SMC should be considered a cost-effective intervention in the Sahelian context.^23,24^

This trial builds on several previous studies in Southern Province, Zambia, including a previous randomized controlled trial of a mass testing and treatment intervention for malaria prevention.^8^ The results here indicate that the intervention may be slightly less cost-effective than mass testing and treatment; however, this result was largely due to higher estimates of cost which themselves were largely due to the use of a more expensive drug and the implementation in a trial context. Furthermore, although attempts were made to ensure that cost and effect modeling used in this study were similar to that used in the previous study, effect estimates and cost estimates may not be compatible.

The cost-effectiveness estimates included in this study are incremental to the use of an enthusiastically implemented package of standard malaria control interventions including long-lasting insecticide-treated bed nets, IRS, and investments in improved case management at health facilities and within communities thorough the scale-up of community health workers equipped with RDTs and artemisinin combination therapies. As such, direct comparison of these results to previous cost-effectiveness estimates of LLIN alone (i.e., versus doing nothing) would be misleading. The do-nothing comparison (generalized cost-effectiveness) is a standard WHO practice but can pose challenges to compare results from cost-effectiveness studies conducted in realistic situations where a do-nothing comparator is seldom available.^21^ Nevertheless, the cost-effectiveness results from this study do not suggest that MDA (or fMDA) would be an intervention that could cost-effectively replace the SoC malaria control interventions, given the much higher cost per person year of protection than LLIN programs can achieve.^26^ Challenges with external comparison to other results notwithstanding the internal validity of this study’s conclusions, suggesting that MDA is superiorly cost-effective (and effective) than fMDA is convincing.

This study used a limited provider perspective and as such did not consider the household costs or cost savings that might arise because of the interventions. It also considered only gross provider costs, meaning that direct cost savings to the health system due to averted treatment were also not included. Although these decisions result in biased estimates of the net cost of these interventions from a societal perspective, they would not have altered the decision analysis results contained in this study. Cost savings from averted treatment either to HH or to the health system would have been expected to be more substantial in the more effective intervention arm, thus increasing the advantage of MDA over fMDA from a cost and cost-effectiveness perspective. Household costs to receive the intervention were expected to be substantially similar in the two interventions or perhaps even lower in the MDA arm, given the lack of need for participants to subject themselves to RDT testing and associated discomfort and time. Another major limitation of this study was that the assessment of deaths and DALYs averted was through simple assumptions about the case fatality rate and the number of DALYs from each subsequent death. Because these assumptions have a direct linear effect on cost per death and cost per DALY, they would have no effect on the decision analysis in this context, as there is no reason to expect the mortality profile to differ between fMDA and MDA. They could result in substantial differences between this study and estimates of cost-effectiveness in other literature which uses different assumptions. Comparisons of the cost per DALY averted to WHO thresholds could also be substantially affected by the choice of parameters for the DALY calculation, however, because the relationship of these parameters to the total number of DALYs is linear and the expected consequences are obvious (i.e., higher case fatality rates mean lower costs per DALY averted and more DALYs per death [longer assumed life expectancies] mean lower cost per DALY averted). Furthermore, the WHO thresholds are somewhat arbitrary as these are not linked directly to the empirical evidence of willingness to pay (WTP) either among Zambian HH or individuals nor are they directly derived from budget analysis of available funds and decision-maker priorities in Zambia. In fact, it has been postulated that the likely supply side thresholds may be substantially lower than the suggested WHO levels which are more likely to correspond to the demand side WTP.^27^

One major concern with the use of MDA or fMDA in endemic malaria settings is the possibility for the emergence or spread of drug resistance to the drug used due to the extensive selection pressure that is being applied to the parasite population during MDA rounds.^28^ Although evidence links indirect MDA, such as chloroquinization of salt, to drug resistance development, it has not been explicitly linked to direct MDA, although MDA might be expected to enhance the spread of already existing drug resistance.^28–31^ Less is known about how fMDA might exacerbate or mitigate this risk of resistance emergence and spread as compared with MDA; however, given the much smaller amounts of drugs distributed (while still using therapeutic doses), it might mitigate the risk of drug resistance development and spread compared with MDA.^28,31^ Although this study and an accompanying parasite clearance study gave no indication of resistance in the study area, the risk of future development of drug resistance in the current first line class of antimalarials is a serious risk of this strategy that must be considered alongside the cost-effectiveness results. Development of drug resistance due to the use of this strategy could seriously bias the long-term accuracy of the cost-effectiveness results presented here; differential development of resistance between fMDA and MDA could also change the conclusions of this study.

Significant declines in parasite prevalence and incidence due to exogenous factors occurred in all trial arms in this study.^19,20^ These changes were believed to be mainly attributable to the scale-up of other malaria control interventions including IRS, expanded case management in the study areas, and changes in weather. Although the large drops in parasite prevalence should not change the effect sizes derived from the trial and used in this study, they are likely to influence the absolute numbers of infections, cases, and deaths estimated to be averted due to the MDA intervention and as such to reduce the cost-effectiveness of these interventions in this setting.

Mass drug administration is an effective strategy for the prevention of malaria in Southern Province, Zambia, and appears to meet the WHO criteria to be considered a cost-effective intervention in the country.
